# Tobacco smoking: Health impact, prevalence, correlates and interventions

**DOI:** 10.1080/08870446.2017.1325890

**Published:** 2017-05-28

**Authors:** Robert West

**Affiliations:** ^a^ Department of Behavioural Science and Health, University College London, London, UK

**Keywords:** smoking, tobacco, addiction

## Abstract

***Background and objectives***: Despite reductions in prevalence in recent years, tobacco smoking remains one of the main preventable causes of ill-health and premature death worldwide. This paper reviews the extent and nature of harms caused by smoking, the benefits of stopping, patterns of smoking, psychological, pharmacological and social factors that contribute to uptake and maintenance of smoking, the effectiveness of population and individual level interventions aimed at combatting tobacco smoking, and the effectiveness of methods used to reduce the harm caused by continued use of tobacco or nicotine in some form.

***Results and conclusions***: Smoking behaviour is maintained primarily by the positive and negative reinforcing properties of nicotine delivered rapidly in a way that is affordable and palatable, with the negative health consequences mostly being sufficiently uncertain and distant in time not to create sufficient immediate concern to deter the behaviour. Raising immediate concerns about smoking by tax increases, social marketing and brief advice from health professionals can increase the rate at which smokers try to stop. Providing behavioural and pharmacological support can improve the rate at which those quit attempts succeed. Implementing national programmes containing these components are effective in reducing tobacco smoking prevalence and reducing smoking-related death and disease.

## Introduction

The continued popularity of tobacco smoking appears to defy rational explanation. Smokers mostly acknowledge the harm they are doing to themselves and many report that they do not enjoy it – yet they continue to smoke (Fidler & West, 2011; Ussher, Brown, Rajamanoharan, & West, 2014). The reason is that nicotine from cigarettes generates strong urges to smoke that undermine and overwhelm concerns about the negative consequences of smoking, and the resolve not to smoke in those trying to stop (West & Shiffman, 2016). Progress is being made in many countries in reducing smoking prevalence but it remains one of the main causes of ill health and premature death worldwide (Gowing et al., 2015).

This paper provides a broad overview of smoking in terms of: the health effects, benefits of stopping, prevalence and patterns of use, psychological, pharmacological and social factors leading to uptake and maintenance of the behaviour, effectiveness of population level and individual level interventions to combat it, and methods used to reduce the harm despite continued use of tobacco or nicotine.

## Definitions of smoking and smoking cessation

Tobacco smoking consists of drawing into the mouth, and usually the lungs, smoke from burning tobacco (West & Shiffman, 2016). The type of product smoked is most commonly cigarettes, but can also include cigarillos, cigars, pipes or water pipes. ‘Smokeless’ tobacco is also popular in some parts of the world. This typically involves using tobacco preparations for chewing, sniffing into the nose or placing as a wad in the mouth between the cheeks and gums (Critchley & Unal, 2003). Smokeless tobacco use has features that are similar to smoking and can carry significant health risks (Critchley & Unal, 2003); however, this article focuses on smoked tobacco only as this has been the subject of by far the largest volume of research and is the most harmful form of tobacco use.

Stopping smoking usually involves an intention not to smoke any more cigarettes from a given point in time (a ‘quit attempt’), followed by self-conscious resistance of urges to smoke resulting in a period of abstinence. If someone making a quit attempt smokes one or more cigarettes on an occasion but then resumes abstinence, this is usually termed a ‘lapse’. If this person resumes smoking on a regular basis s/he is said to have ‘relapsed’. ‘Short-term abstinence’ is commonly defined in terms of achieving up to 4 weeks of abstinence. ‘Long-term abstinence’ often refers to abstinence for at least 6 months but more typically involves abstinence for at least 12 months. There is no agreed criterion for deciding when someone has ‘stopped smoking’ so it is essential when using the term to be clear about how long the abstinence period has been.

## Health impact of smoking and the benefits of stopping

Tobacco smoking increases the risk of contracting a wide range of diseases, many of which are fatal. Stopping smoking at any age is beneficial compared with continuing to smoke. For some diseases, the risk can be reversed while for others the risk is approximately frozen at the point when smoking stopped.

### Health impact of smoking

Table 1 lists the main causes of death from smoking. Tobacco smoking is estimated to lead to the premature death of approximately 6 million people worldwide and 96,000 in the UK each year (Action on Smoking and Health, 2016b; World Health Organization, 2013). A ‘premature death from smoking’ is defined as a death from a smoking-related disease in an individual who would otherwise have died later from another cause. On average, these premature deaths involve 10 years of life years lost (US Department of Health and Human Services, 2004). Many of these deaths occur in people who have stopped smoking but whose health has already been harmed by smoking. It also happens to be the case that smokers who do not stop smoking lose an average of 10 years of life expectancy compared with never-smokers and they start to suffer diseases of old age around 10 years earlier than non-smokers (Jha & Peto, 2014).

**Table 1.** Main causes of death from tobacco smoking and benefits of stopping.

Cause of death from smoking	Benefit of stopping smoking
Coronary heart disease and stroke	Preventable if cessation occurs in early adulthood; at least partially reversible thereafter
Cancers of the lung and upper airways	Preventable if cessation occurs in early adulthood; further increase in risk prevented thereafter
Chronic obstructive pulmonary disease	Preventable if cessation occurs in early adulthood; further decline in lung function slowed thereafter
Miscarriage and underdevelopment of foetus	Preventable if cessation occurs early in pregnancy; risk is mitigated by stopping at any time in pregnancy

Most smoking-related deaths arise from cancers (mainly lung cancer), respiratory disease (mainly chronic obstructive pulmonary disease – COPD), and cardiovascular disease (mainly coronary heart disease) (Action on Smoking and Health, 2016b). Smoking is an important risk factor for stroke, blindness, deafness, back pain, osteoporosis, and peripheral vascular disease (leading to amputation) (US Department of Health and Human Services, 2004). After the age of 40, smokers on average have higher levels of pain and disability than non-smokers (US Department of Health and Human Services, 2004).

Smoking in both women and men reduces fertility (Action on Smoking and Health, 2013). Smoking in pregnancy causes underdevelopment of the foetus and increases the risk of miscarriage, neonatal death, respiratory disease in the offspring, and is probably a cause of mental health problems in the offspring (Action on Smoking and Health, 2013).

People used to think that smoking was protective against Alzheimer’s disease but we now know that the opposite is the case: it is a major risk factor for both Alzheimer’s and vascular dementia (Ferri et al., 2011; US Department of Health and Human Services, 2004).

There is a positive association between average daily cigarette consumption and risk of smoking-related disease, but in the case of cardiovascular disease the association is non-linear, so that low levels of cigarette consumption carry a higher risk than would be expected from a simple linear relationship (US Department of Health and Human Services, 2004).

Tobacco smoke contains biologically significant concentrations of known carcinogens as well as many other toxic chemicals. Some of these, including a number of tobacco-specific nitrosamines (particularly NNK and NNN) are constituents of tobacco, largely as a result of the way it is processed, while others such as benzopyrine result from combustion of tobacco (Action on Smoking and Health, 2014b). These chemicals form part of the particulate matter in smoke. Tobacco smoke also contains the gas, carbon monoxide (CO). CO is a potent toxin, displacing oxygen from haemoglobin molecules. However, acutely the amount of CO in tobacco smoke is too small to lead to hypoxia and the body produces increased numbers of red blood cells to compensate.

The nicotine in tobacco smoke may cause a small part of the increase in cardiovascular disease but none or almost none of the increase in risk of respiratory disease or cancer (Benowitz, 1997, 1998). It is the other components of cigarette smoke that do almost all the damage. It has been proposed on the basis of studies with other species that nicotine damages the adolescent brain but there is no evidence for clinically significant deficits in cognition or emotion in adults who smoked during adolescence and then stopped (US Department of Health and Human Services, 2004).

Exposure to second-hand smoke carries a significant risk for both children and adults. Thus, non-smokers who are exposed to a smoky environment have an increased risk of cancer, heart disease and respiratory disease (Action on Smoking and Health, 2014a).

### Benefits of stopping smoking

Table 1 lists the main benefits of stopping smoking. Smokers who stop before their mid-30s have approximately the same life expectancy as never smokers (Doll, Peto, Boreham, & Sutherland, 2004; Pirie, Peto, Reeves, Green, & Beral, 2013). After the age of 35 years or so, stopping smoking recovers 2–3 months of healthy life expectancy for every year of smoking avoided, or 4–6 h for every day (Jha & Peto, 2014).

Stopping smoking has different effects on different smoking-related diseases. Excess risk of heart attack caused by smoking reduces by 50% within 12 months of stopping smoking. Stopping smoking returns the rate of decline in lung function to the normal age-related decline, but does not reverse this; it reduces the frequency of ‘exacerbations’ (acute attacks of breathing difficulty resulting in death or hospitalisation) in COPD patients (US Surgeon General, 1990). Stopping smoking ‘freezes’ the risk of smoking-related cancers at the level experienced when stopping occurs but does not decrease it in absolute terms (US Surgeon General, 1990).

Smokers who stop show reduced levels of stress and mood disorder than those who continue (Royal College of Physicians and Royal College of Psychiatrists, 2013). They also report higher levels of happiness and life satisfaction than those who continue (Shahab & West, 2009, 2012). This suggests that smoking may harm mental health, though other explanations cannot be ruled out on the current evidence.

## Prevalence and patterns of smoking

### Smoking prevalence

There are estimated to be approximately 1 billion tobacco smokers worldwide (Eriksen, Mackay, & Ross, 2013), amounting to approximately 30% of men and 7% of women (Gowing et al., 2015).

Cigarette smoking prevalence in Great Britain was estimated to be 16.9% in 2015, the most recent year for which figures are available at the time of writing: slightly lower in women than men (Office of National Satistics, 2016). Smoking in Great Britain has declined by 0.7 percentage points per year since 2001 (from 26.9% of adults in 2001). In Australia, daily cigarette smoking has declined by 0.6 percentage points per year over a similar time period (from 22.4% of adults aged 18 + years in 2001 to 14.5% in 2015) (Australian Bureau of Statistics, 2015). However, international comparisons are confused by different countries using a different definition of what counts as being a smoker, and different methods for assessing prevalence. Australia only counts daily smokers in their headline figures. The situation in the US is even more misleading. The headline prevalence figure for the US is below 16%, but this does not include occasional smokers and people who smoke cigarillos which are essentially cigarettes in all but name and which have become increasingly popular in recent years. So the figure for prevalence that is most comparable to the figure for Great Britain is 20% (Jamal, 2016).

With the above caveats in mind, the figures in Table 2 for smoking prevalence in world regions in men and women provide very broad estimates (Gowing et al., 2015). Most noteworthy is that smoking prevalence in men is more than four times that in women globally but that the difference is much less in most parts of Europe, and that Eastern Europe as a whole has the highest smoking prevalence of any region in the world.

**Table 2.** Estimates of tobacco smoking prevalence in world regions.

Region	Male prevalence %	Female prevalence %	Overall prevalence %
Africa	23	3	13
Caribbean Central and Northern America	20	4	13
South America	30	15	21
Central Southern and Western Asia	37	4	23
Eastern and South-eastern Asia	45	4	24
Eastern Europe	42	22	31
Northern Europe	28	22	27
Southern Europe	35	24	28
Western Europe	33	24	29
Oceania	43	19	30
World	32	7	23

Note: Current smoking of any tobacco product, adults aged 15 years and older, age-standardised rate, by gender. ‘Tobacco smoking’ includes cigarettes, cigars, pipes or any other smoked tobacco products. ‘Current smoking’ includes both daily and non-daily or occasional smoking. From Gowing et al. (2015).

### Smoking patterns

The most common age of first trying a cigarette in countries that have been studied is 10–15 years (Action on Smoking and Health, 2015b; Talip, Murang, Kifli, & Naing, 2016); take up of regular smoking usually continues up to early 20s (Dierker et al., 2008).

Average daily cigarette consumption among smokers in the US and UK has declined steadily since the 1970s. In the UK, it is currently 11 cigarettes per day, and non-daily smoking is very rare (Action on Smoking and Health, 2016c; Jarvis, Giovino, O’Connor, Kozlowski, & Bernert, 2014). Smokers take in an average of 1–1.5 mg of nicotine per cigarette (US Department of Health Human Services, 2014). The US figures on patterns of smoking are distorted by not counting ‘cigarillos’ and other smoked tobacco products which are used very much like cigarettes, whose prevalence has increased in recent years (Jamal et al., 2015). The reduction in daily cigarette consumption has not been accompanied by a reduction in daily nicotine intake (Jarvis et al., 2014). This could be due to the use of other smoked tobacco products (in the case of the US) or smokers smoking their cigarettes more intensively (taking more, deeper or longer puffs).

Smokers in England spend an average of £23 per week on cigarettes and this figure is slowly rising (West & Brown, 2015). In the UK, hand-rolled cigarettes have become increasingly popular with 34% of smokers currently reporting use of these products (Action on Smoking and Health, 2016c). Men and people in more deprived socio-economic groups are more likely to smoke hand-rolled cigarettes (Action on Smoking and Health, 2016c).

In most countries, there are strong negative associations between smoking prevalence and educational level, affluence and mental health; and positive associations with alcohol use disorder and substance use disorder (Action on Smoking and Health, 2016a, 2016c; Royal College of Physicians and Royal College of Psychiatrists, 2013; Talati, Keyes, & Hasin, 2016). In the UK, average daily cigarette consumption is higher for men than women, and higher in smokers in more deprived socio-economic groups and those with mental health problems (Action on Smoking and Health, 2016c).

## Psychological, pharmacological and social factors involved in smoking and smoking cessation

**Figure 1.**
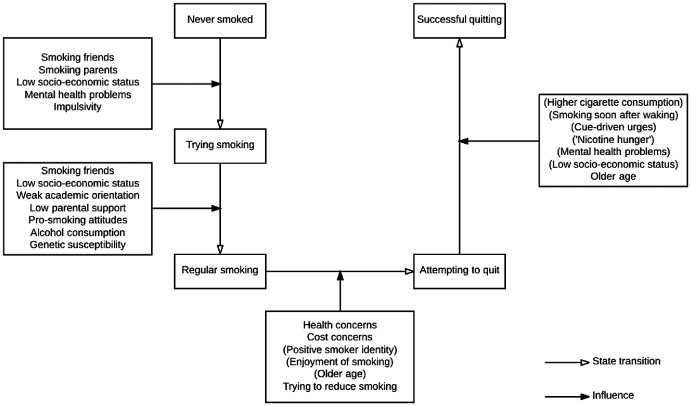
Factors associated with transitions in the natural history of smoking (parentheses indicate negative associations).

The natural history of smoking can be modelled as states and factors that influence the transition between these. Figure 1 shows transitions that have been researched – the variables identified in the diagram are listed descriptively without attempting to explain how they may be connected.

### Smoking initiation

Important factors predicting initiation in western societies are: having friends who smoke, having parents who smoke, low social grade, tendency to mental health problems and impulsivity (Action on Smoking and Health, 2015b). Transition to daily smoking follows a highly variable pattern sometimes being very rapid and sometimes taking several years (Schepis & Rao, 2005). Important factors predicting transition to regular smoking are: having friends who smoke, weak academic orientation, low parental support, pro-smoking attitudes, drinking alcohol and low socio-economic status (Action on Smoking and Health, 2015b).

Smoking initiation has a ‘heritability’ (the proportion of variance in a characteristic that is attributable to genetic rather than environmental variance) of approximately 30–50% in western societies (Vink, Willemsen, & Boomsma, 2005). This means that differences in genetic make-up account for almost half of the difference in likelihood of starting smoking between individuals. This does not mean that environmental factors do not also play a crucial role as is evident from the very large decline in smoking initiation since the 1970s in many western countries.

The heritability of cigarette addiction (as distinct from smoking) is approximately 70–80% in western societies (Vink et al., 2005). Cigarette addiction here refers to the extent to which someone experiences a strong need to smoke. It is usually indexed by a combination of number of cigarettes per day and time from waking to smoking the first cigarette of the day (Kozlowski, Porter, Orleans, Pope, & Heatherton, 1994). It can also be indexed by the self-reported strength of urges to smoke (Fidler, Shahab, & West, 2011). Heritability of cigarette addiction, as indexed by failure of attempts to stop, is higher than the heritability for smoking and for initiation of smoking. This suggests that differences in genetic inheritance play a larger role in being able to stop smoking than in starting to smoke.

### Cigarette addiction

Cigarette addiction stems from the fact that smoking provides highly controllable doses of the drug, nicotine, rapidly to the brain in a form that is accessible, affordable and palatable (West, 2009; West & Shiffman, 2016). Nicotine provided more slowly, for example by the nicotine transdermal patch, is much less addictive. It is possible that one or more mono-amine oxidase inhibitors in cigarette smoke add to, or synergise, the addictive properties of nicotine (Hogg, 2016).

The psychopharmacology of cigarette addiction is complex and far from fully understood. The following paragraphs summarise the current narrative.

Nicotine resembles the naturally occurring neurotransmitter, acetylcholine, sufficiently to attach itself to a subset of neuronal receptors for this neurotransmitter in the brain. These are called ‘nicotinic acetylcholine receptors’. When it does this with receptors in the ventral tegmental area in the midbrain, it causes an increased rate of firing of the nerves projecting forward from that area to another part of the brain called the nucleus accumbens. This causes release of another neurotransmitter called dopamine in the nucleus accumbens.

Dopamine release and uptake by neurones in the nucleus accumbens is believed to be central to all addictive behaviours. It acts as a neural ‘teaching signal’ which causes the brain to form an association between the current situation as perceived and the impulse to engage in whatever action immediately preceded this release. In the case of smoking, this creates an urge to smoke in situations in which smoking frequently occurs. These are often referred to as ‘cue-driven smoking urges’ or ‘situational cravings’ (West, 2009; West & Shiffman, 2016). This explains why even non-daily smokers often find it difficult to stop smoking altogether.

Repeated ingestion of nicotine from cigarettes causes changes to the functioning of the ventral tegmental area and nucleus accumbens such that when brain concentrations of nicotine are lower than usual, there is an abnormally low level of neural activity in these regions. This leads to feelings of need for behaviours that have in the past restored normal functioning, typically smoking. This feeling of need can be thought of as a kind of ‘nicotine hunger’, also called ‘background craving’ (West, 2009; West & Shiffman, 2016). This is probably why time between waking and first cigarette of the day is a useful predictor of difficulty stopping smoking (Vangeli, Stapleton, Smit, Borland, & West, 2011). So ‘cue-driven smoking urges’ and ‘nicotine hunger’ are important factors contributing to smoking behaviour and thought to be the primary mechanisms underpinning cigarette addiction (West, 2009; West & Shiffman, 2016).

When smokers abstain from cigarettes, within a few hours many of them start to experience nicotine withdrawal symptoms. Withdrawal symptoms from a drug are temporary symptoms that arise when the drug dose is reduced or use is terminated. They arise from neural adaptation to the presence of the drug in the central nervous system. For smoking, the most common early onset symptoms are: irritability, restlessness and difficult concentrating. Depression and anxiety have also been observed in some smokers. These symptoms typically last 1 to 4 weeks (West, 2009; West & Shiffman, 2016).

After a day or two of stopping smoking, many smokers experience other symptoms: increased appetite, constipation, mouth ulcers, cough, and weight gain. Increased appetite tends to last for at least 3 months; weight gain (averaging around 6 kg) tends to be permanent; other symptoms tend to last a few weeks. The increased appetite, weight gain and constipation arise from termination of nicotine intake but the others are probably related to other effects of stopping smoking (West, 2009; West & Shiffman, 2016).

Any of the above effects of abstinence may in individual cases promote resumption of smoking following a quit attempt but statistically the association is inconsistent and weak; the main factors driving relapse appear to be cue-driven smoking urges and nicotine hunger (Fidler & West, 2011; West, 2009; West & Shiffman, 2016).

Many smokers report that smoking helps them cope with stress and increases their ability to concentrate. However, this appears to be because when they go for a period without smoking they experience nicotine withdrawal symptoms that are relieved by smoking. Long-term smokers who stop report lower levels of stress than when they were smoking and no reduction in ability to concentrate (West, 2009; West & Shiffman, 2016).

It is commonly thought that smokers with mental health problems are using cigarettes to ‘self-medicate’ or treat their psychological symptoms. However, the evidence indicates that neither nicotine nor smoking improves psychological symptoms, and people with serious mental health disorders who stop smoking do not experience a worsening of mental health. In fact some studies have found an improvement (Royal College of Physicians and Royal College of Psychiatrists, 2013).

### Smoking cessation

For most smokers, cessation requires a determined attempt to stop and then sufficient resolve in the following weeks and months to overcome what are often powerful urges to smoke. Factors that predict quit attempts differ from those that predict the success of those attempts (Vangeli et al., 2011). Approximately 5% of unaided quit attempts succeed for at least 6 months (Hughes, Keely, & Naud, 2004). Relapse after this point is estimated to be around 50% over subsequent years (Stapleton & West, 2012).

The most common self-reported reasons for smoking are stress relief and enjoyment, with around half of smokers reporting these smoking motives. Weight control, aiding concentration and socialising are also quite commonly cited (Fidler & West, 2009). Smoking for supposed stress relief, improved concentration, weight control or other functions has not been found to be related to attempts to stop or success of attempts to stop (Fidler & West, 2009). Smokers who report enjoying smoking are less likely to try to stop but not less likely to succeed if they do try (Fidler & West, 2011). In addition, having a positive smoker identity (liking being a smoker) predicts not trying to quit, over and above enjoyment of smoking (Fidler & West, 2009).

No clear association has been found between the number of times smokers have tried to stop in the past and their chances of success the next time they try (Vangeli et al., 2011). However, having tried to stop *in the past few months* is predictive of failure of the next quit attempt (Zhou et al., 2009). Belief in the harm caused by smoking is predictive of smokers making quit attempts but not the success of those attempts (Vangeli et al., 2011).

Some clinical studies have found that women were less likely to succeed in quit attempts than men but large population studies have found no difference in success rates between the genders (Vangeli et al., 2011) so it may be the case that women who seek help with stopping have greater difficulty than men who seek help with stopping.

Number of cigarettes smoked per day, time between waking and the first cigarette of the day and rated strength of urges to smoke prior to a quit attempt have been found to predict success of quit attempts (Vangeli et al., 2011).

Quit attempts that involve gradual reduction are less likely to succeed than those that involve quitting abruptly, even after controlling statistically for measures of cigarette addiction, confidence in quitting, other methods used to quit (e.g. nicotine replacement therapy) and sociodemographic factors (Lindson-Hawley et al., 2016).

## Interventions to combat smoking

There is extensive evidence on interventions that can reduce smoking prevalence, either by reducing initiation or promoting cessation. Table 3 lists those that have the strongest evidence.

**Table 3.** Effective interventions for combating smoking.

Intervention	Effectiveness
Increasing the financial cost through increasing excise duty and reducing illicit supply	1–2 percentage point reduction in prevalence for 10% increase in cost of smoking; increases cessation and reduces initiation
Anti-tobacco marketing campaigns	Effect on cessation and initiation varies with content and intensity of campaigns
Brief physician advice to smokers	1–3 percentage point increase in long-term smoking cessation rate in all those receiving it regardless of initial motivation to quit
Prescription for varenicline, nicotine replacement therapy, bupropion, nortriptyline or cytisine	5–15 percentage point increase in quit success in those using it to try to quit (highest with varenicline and nicotine patches plus faster acting nicotine replacement therapy)
Behavioural support, either face to face or by telephone	3–10 percentage point increase in long-term quit success among those using it to try to quit for multi-session support delivered by trained specialists, the effect apparently being additive with pharmacotherapy
Printed self-help materials	1–2 percentage point increase in long-term quit success in those using it to try to quit compared with nothing
Peer-led school-based anti-smoking programmes and social competence training	Reduction in youth uptake varies with content and intensity of the programme

### Population-level interventions

Increasing the financial cost of smoking through tax increases and control of illicit supply on average reduces overall consumption with a typical price elasticity globally of 0.4 (meaning that for every 10% increase in the real cost there is a 4% decrease in the number of cigarettes purchased). Most of the effect is in getting smokers to reduce their daily cigarette consumption so the effect on smoking prevalence has been found to be an average of a 1–2 percentage point prevalence reduction for every 10% increase in the real cost (Levy, Huang, Havumaki, & Meza, 2016). It has been claimed that increasing taxes on tobacco increases the amount of smuggling of cheap tobacco, but the evidence does not support this (Action on Smoking and Health, 2015a; Joossens & Raw, 2003).

Social marketing campaigns (e.g. TV advertising) can prevent smoking uptake, increase the rate at which smokers try to quit and improve the chances of success. This can lead to a reduction in smoking prevalence. Their effectiveness varies considerably with intensity, type of campaign and context (Bala, Strzeszynski, Topor-Madry, & Cahill, 2013; Hoffman & Tan, 2015).

Legislating to ban smoking in all indoor public areas may have a one-off effect on reducing smoking prevalence but findings are inconsistent across different countries (Bala et al., 2013). For example, in countries such as France it was not possible to detect an effect while in England, there did appear to be a decline in prevalence following the ban.

Although it is hard to show conclusively, circumstantial evidence suggests that banning tobacco advertising and putting large graphic health warnings on cigarette packets may have reduced smoking prevalence in some countries (Hoffman & Tan, 2015; Noar et al., 2016).

### Individual-level interventions to promote smoking cessation

#### Brief advice

Brief advice to stop smoking from a physician and offer of support to all smokers, regardless of motivation to quit, has been found in randomised trials to increase rate of quitting by an average of 2 percentage points of all those receiving it, whether or not they were initially interested in quitting (Stead et al., 2013). The offer of support appears to be more effective in getting smokers to try to quit than just advising smokers to stop (Aveyard, Begh, Parsons, & West, 2012).

#### Pharmacotherapy

Using a form of nicotine replacement therapy (NRT: transdermal patch, chewing gum, nasal spray, mouth spray, lozenge, inhalator, dissolvable strip) for at least 6 weeks from the start of a quit attempt increases the chances of long-term success of that quit attempt by about 3–7 percentage points if the user is under the care of a health professional or provided as part of a structured support programme (Stead et al., 2012). Some studies have found that NRT when bought from a shop and used without any additional structured support does not improve the chances of success at stopping (Kotz, Brown, & West, 2014a, 2014b). A small proportion of people who use NRT to stop smoking continue to use it for months or even years after stopping smoking, but NRT appears to carry minimal risk to long-term users (Royal College of Physicians, 2016; Stead et al., 2012).

Data are sparse but at present, using an electronic cigarette in a quit attempt appears to increase the chances of success at stopping on average by an amount broadly similar to that from NRT; the variety of products available and the greater similarity to smoking appear to make them more attractive to many smokers as a means of stopping than NRT (McNeill et al., 2015; Royal College of Physicians, 2016). Electronic cigarettes deliver nicotine to users by heating a liquid containing nicotine, propylene glycol or glycerol and usually flavourings to create a vapour that is inhaled. They appear to carry minimal acute risk to users. If they are used long-term, their risk is almost certainly much less than that of smoking (based on concentrations of chemicals in the vapour) (McNeill et al., 2015; Royal College of Physicians, 2016).

‘Dual-form NRT’ (combining a transdermal NRT patch and one of the other forms) increases the chances of success at stopping more than ‘single-form NRT’ (just using one of the products) (Stead et al., 2012). Starting to use a nicotine transdermal patch several weeks before the target quit date may improve the chances of success at quitting compared with starting on the quit date (Stead et al., 2012).

Taking the prescription anti-depressant, bupropion (brand name Zyban), improves the chances of success of quit attempts by a similar amount to single-form NRT (Hughes, Stead, Hartmann-Boyce, Cahill, & Lancaster, 2014). Bupropion often leads to sleep disturbance and carries a very small risk of seizure. Bupropion probably works by reducing urges to smoke rather than any effect on depressed mood, but how it does this is not known. It is contra-indicated in pregnant smokers and people with an elevated seizure risk or history of eating disorder (Hughes et al, 2014). Taking the tricyclic anti-depressant, nortriptyline also improves the chances of success of quit attempts, probably by about the same amount as bupropion and NRT (Hughes et al., 2014). Its mechanism of action is not known. Nortriptyline often leads to dry mouth and sleep disorder and can be fatal in overdose (Hughes et al., 2014).

Taking the nicotinic-acetylcholine receptor partial agonist, varenicline (brand name Chantix in the US and Champix elsewhere), improves the chances of success by about 50% more than bupropion or single-form NRT (Cahill, Lindson-Hawley, Thomas, Fanshawe, & Lancaster, 2016). This is true for smokers with or without a psychiatric disorder (Anthenelli et al., 2016). Varenicline appears to work both by reducing urges to smoke and the rewarding effect of nicotine should a lapse occur (West, Baker, Cappelleri, & Bushmakin, 2008). Varenicline often leads to sleep disturbance and nausea. Serious neuropsychiatric and cardiovascular adverse reactions have been reported, but in comparative studies these have not been found to be more common than placebo or NRT (Anthenelli et al., 2016; Cahill et al., 2016; Sterling, Windle, Filion, Touma, & Eisenberg, 2016).

Taking the nicotinic-acetylcholine receptor partial agonist, cytisine, appears to improve the chances of success at least as much as single-form NRT and probably more (Cahill et al., 2016). Cytisine often causes nausea. No serious adverse reactions have been reported to date (Cahill et al., 2016). Where it is licensed for sale, cytisine is less than 1/10th the cost of other smoking cessation medications (Cahill et al., 2016).

#### Behavioural support

There is good evidence that behavioural interventions of many kinds, delivered though several modalities can help smokers to stop. Thus, behavioural support (encouragement, advice and discussion) from a trained stop-smoking specialist, provided at least weekly until at least 4 weeks following the target quit date can increase the chances of long-term success of a quit attempt by about 3–7 percentage points, whether it is given by phone or face-to-face (Lancaster & Stead, 2005). Group behavioural support (specialist-led groups of smokers stopping together and engaging in a structured discussion about their experiences), involving at least weekly sessions lasting until at least 4 weeks after the target quit date can increase the chances of success of a quit attempt by a similar amount or possibly more than individual support (Stead & Lancaster, 2005). Scheduled, multi-session telephone support can improve rates of success at stopping smoking by a broadly similar amount (Stead, Hartmann-Boyce, Perera, & Lancaster, 2013) but some large studies have failed to detect an effect so contextual factors and/or the precise type of support could be crucial to success. The effects of behavioural support and medication/NRT on success at stopping smoking appear to combine roughly additively (Stead, Koilpillai, & Lancaster, 2015). Smoking cessation support appears to be effective in primary care, secondary care and worksite settings (Cahill & Lancaster, 2014; West et al., 2015). Financial incentives, in the form of vouchers, have been found to increase smoking cessation rates for as long as they are in place (Cahill, Hartmann-Boyce, & Perera, 2015; Higgins & Solomon, 2016). Printed self-help materials can improve the chances of success at stopping long term by around 1–2 percentage points (Hartmann-Boyce, Lancaster, & Stead, 2014).

There is still relatively limited evidence on the effectiveness of digital support interventions for smoking cessation. Thus, while there is evidence that tailored, interactive websites can improve the chances of success at stopping smoking compared with no support, brief written materials or static information websites, many of those tested have not been found to be effective and it is not clear what differentiates those that are effective from those that are not (Graham et al., 2016). Text messaging programmes have been found to increase the chances of success of quit attempts by about 2–7 percentage points (Whittaker, McRobbie, Bullen, Rodgers, & Gu, 2016). There is currently insufficient evidence to know whether smartphone applications can improve success rates of quit attempts, although preliminary data suggest that they might (Whittaker et al., 2016). Evidence on alternative and complementary therapies is not sufficient to make confident statements about their effectiveness as aids to smoking cessation (Barnes et al., 2010; White, Rampes, Liu, Stead, & Campbell, 2014).

Overall, the highest smoking cessation rates appear to be achieved using specialist face-to-face behavioural support together with either varenicline or dual form NRT. With this support, continuous abstinence rates up to 52 weeks, verified by expired-air carbon monoxide tests, of more than 40% have been achieved (Kralikova et al., 2013). More commonly, 52-week continuous abstinence rates with this treatment are between 15 and 25% (West et al., 2015).

#### Smoking cessation support for pregnant smokers

In pregnant smokers, there is some evidence that NRT can help promote smoking cessation but evidence for an effect sustained to end of pregnancy is not conclusive (Sterling et al., 2016). There is also evidence that written self-help materials and face-to-face behavioural support can aid smoking cessation (Jones, Lewis, Parrott, Wormall, & Coleman, 2016), and financial incentives have also been found to improve quitting rates among pregnant smokers (Tappin et al., 2015). Almost half of women who stop smoking during pregnancy as a result of a clinical intervention relapse to smoking within 6 months of the birth (Jones et al., 2016).

### Effectiveness of programmes to reduce smoking uptake

School-based programmes that involve both social competence training and peer-led social influence have been found to reduce smoking uptake (Georgie, Sean, Deborah, Matthew, & Rona, 2016) but educational programmes have not (Thomas, McLellan, & Perera, 2013). Mass media campaigns and increasing the financial cost of smoking reduce smoking uptake (Brinn, Carson, Esterman, Chang, & Smith, 2012; van Hasselt et al., 2015).

### Reducing the harm from tobacco and nicotine use

Smokers who report that they are reducing their cigarette consumption smoke only 1–2 fewer cigarettes per day on average than when they say they are not (Beard et al., 2013). Clinical trials have found that use of NRT while smoking can substantially reduce cigarette consumption compared with placebo (Royal College of Physicians, 2016) but national surveys show very little reduction in cigarette consumption when smokers take up use of NRT in real-world settings (Beard et al., 2013). The benefit from using NRT while continuing to smoke appears to be in promoting subsequent smoking cessation. Using NRT (or varenicline) to reduce cigarette smoking with no immediate plans to quit leads to increased rates of quitting subsequently (Wu, Sun, He, & Zeng, 2015).

‘Snus’, a form of tobacco that is placed between the gums and the cheek and which is prepared in a way that is very low in carcinogens, gives high doses of nicotine but without evidence of an increase in risk of major tobacco-related cancers and either no, or a small, increase in risk of heart disease. It does appear to increase risk of periodontal disease, however. Snus is very popular in Sweden. Sweden has very low rates of smoking and tobacco-related disease indicating that a form of nicotine intake other than smoking can become popular and suggesting that this can contribute to a substantial reduction in tobacco-related harm (Royal College of Physicians, 2016).

The introduction of complete bans on smoking in indoor public areas can also be considered as a harm reduction measure. In this case, the main issue is harm to non-tobacco users. The evidence shows that such bans have been rapidly followed in the UK and several other jurisdictions by a reduction in heart attacks in non-smokers (Action on Smoking and Health, 2014a).

## Conclusions

Tobacco smoking causes death and disability on a huge scale and only about half of smokers report enjoying it. Despite this, approximately 1 billion adults engage in this behaviour worldwide and only around 5% of unaided quit attempts succeed for 6 months or more. The main reason appears to be that cigarettes deliver nicotine rapidly to the brain in a form that is convenient, and palatable. Nicotine acts on the brain to create urges to smoke in situations where smoking would normally occur and when brain nicotine levels become depleted. Concern about the harm from, and financial cost of, smoking are mostly not sufficient to counter this.

Governments can reduce smoking prevalence by raising the cost of smoking through taxation, mounting sustained social marketing campaigns, ensuring that health professionals routinely advise smokers to stop and offer support for quitting, and make available pharmacological and behavioural support for stopping.

## Statement of competing interests

RW has, within the past 3 years, undertaken research and consultancy for companies that develop and manufacture smoking cessation medications (Pfizer, GSK, and J&J). He is an unpaid advisor to the UK’s National Centre for Smoking cessation and Training. His salary is funded by Cancer Research UK.

## Disclosure statement

No potential conflict of interest was reported by the author.

## Funding

This work was supported by Cancer Research UK [grant number C1417/A22962].
